# Factors determining outcome of corrective osteotomy for malunited paediatric forearm fractures: a systematic review and meta-analysis

**DOI:** 10.1177/1753193417711684

**Published:** 2017-06-15

**Authors:** K. C. Roth, M. M. J. Walenkamp, R. C. I. van Geenen, M. Reijman, J. A. N. Verhaar, J. W. Colaris

**Affiliations:** 1 Department of Orthopaedics, Erasmus University Medical Centre, Rotterdam, The Netherlands; 2 Trauma Unit, Department of Surgery, Academic Medical Centre University of Amsterdam, Amsterdam, The Netherlands; 3 Department of Orthopaedics, Amphia Hospital, Breda, The Netherlands

**Keywords:** Corrective osteotomy, malunion, fracture, forearm, radius, child

## Abstract

The aim of this study was to identify predictors of a superior functional outcome after corrective osteotomy for paediatric malunited radius and both-bone forearm fractures. We performed a systematic review and meta-analysis of individual participant data, searching databases up to 1 October 2016. Our primary outcome was the gain in pronosupination seen after corrective osteotomy. Individual participant data of 11 cohort studies were included, concerning 71 participants with a median age of 11 years at trauma. Corrective osteotomy was performed after a median of 12 months after trauma, leading to a mean gain of 77° in pronosupination after a median follow-up of 29 months. Analysis of variance and multiple regression analysis revealed that predictors of superior functional outcome after corrective osteotomy are: an interval between trauma and corrective osteotomy of less than 1 year, an angular deformity of greater than 20° and the use of three-dimensional computer-assisted techniques.

**Level of evidence:** II

## Introduction

Displaced forearm fractures in children are commonly treated by closed reduction and cast immobilization. This treatment carries the risk of re-displacement of the fracture in cast, resulting in malunion (van Geenen and Besselaar, 2007). In general, young children with a malunion located close to the most active distal physis have the potential to remodel and have unrestricted function and a satisfactory cosmetic outcome. However, both-bone forearm fractures localized in the distal metaphysis have a high chance (60%) of developing a clinically relevant limitation of forearm rotation in case of more severe angular malalignment (greater than 16°), whereas children with diaphyseal both-bone forearm fractures have a moderate chance of limitation (13%–33%) irrespective of the severity of the angular malalignment (Colaris et al., 2014a). Unfortunately, severe malunions in older children have less potential for remodelling, which can result in disappointing clinical outcomes. Nevertheless, there is still no consensus on how much angular deformity is acceptable (Hove and Brudvik, 2008; Ploegmakers and Verheyen, 2006; Roth et al., 2014). Although malunions of forearm fractures in children are relatively uncommon, they have a tendency to result in persistent functional impairment (Fuller and McCullough, 1982; Nagy et al., 2008). For these children, a corrective osteotomy may be considered, but few articles have been published on the outcome of corrective osteotomy for malunited forearm fractures in children. Previous studies have found that corrective osteotomies performed in patients older than 10 years and a time from injury until osteotomy of more than 1 year showed less favourable results (Miyake et al., 2012; Trousdale and Linscheid, 1995; van Geenen and Besselaar, 2007). Other studies have indicated that the location and type of fracture, the level of pre-operative disability and use of computed tomography (CT)-based three-dimensional computer-assisted planning techniques may affect functional outcome after corrective osteotomy (Leong et al., 2010; Miyake et al., 2012; Nagy et al., 2008). All previous studies have reported only small numbers of patients, limiting the reliability of the results. The aim of this study was to conduct a meta-analysis of individual participant data (IPD) to provide the best available evidence on determinants of a superior functional outcome after corrective osteotomy for malunited radius or both-bone forearm fractures in children.

## Methods

We performed a meta-analysis of IPD, which we reported according to the Preferred Reporting Items for Systematic Reviews and Meta-Analysis of Individual Participant Data (PRISMA-IPD) statement (Stewart et al., 2015). Prior to starting the systematic search, we defined the research question, inclusion and exclusion criteria, treatment of interest and outcomes of interest. The protocol of this meta-analysis can be accessed on PROSPERO with trial registration number: PROSPERO CRD42015023964.

We included prospective and retrospective cohort studies containing data on functional outcomes (raw data published or supplied on request). Eligible participants were children with post-traumatic malunion of the radius or both forearm bones, who underwent a corrective osteotomy because of impairment in pronosupination. Patients with an age at trauma of 16 years or younger; an age at corrective osteotomy of 18 years or younger; and an interval between trauma and corrective osteotomy of greater than 6 weeks, were included. We excluded participants with complex fractures (Monteggia, Galeazzi, intra-articular or open fractures) and those treated by callus osteoclasis. Our treatment of interest was corrective osteotomy, subdividing conventional corrective osteotomies using two-dimensional radiographic planning and CT-based three-dimensional computer-assisted corrective osteotomies. Our primary outcome of interest was the gain in forearm rotation measured at final follow-up after corrective osteotomy. Minimum follow-up required was 6 months after corrective osteotomy. Factors possibly influencing the gain in range of motion (ROM) observed after corrective osteotomy were analysed. Data were sought for the following variables: age at injury; age at osteotomy; time from trauma until osteotomy; level of malunion; single or both-bone fracture; degree of angular deformity; and the use of three-dimensional computer-assisted techniques.

To identify all studies regarding the outcome after corrective osteotomy for post-traumatic malunions of the forearm in children, the following databases were searched: Medline, Embase, Web-of-Science, Scopus, Cinahl, Pubmed publisher, Cochrane and Google Scholar for articles published before 21 March 2016. We repeated the search on the 1 October 2016. The complete search strategy is described in Appendix 1 (available online). The search was limited to articles written in English, Dutch or German. Two reviewers (KCR and JWC) assessed the studies for relevance by initially reviewing the titles and abstracts and categorizing the articles in folders of relevancy within an EndNote library. All studies containing functional outcomes after corrective osteotomy of the radius or forearm were deemed potentially relevant. Next, the full manuscript was retrieved to determine appropriateness, by verifying if the studies met the inclusion and exclusion criteria. Any disagreements were resolved by consensus or consultation of a third reviewer. The references of the retrieved studies were scanned to identify additional relevant publications missed by the initial search.

The included studies were evaluated for their methodological quality by two authors (KCR and MMJW) independently. The Methodological Index for Non-Randomized Studies score (MINORS) was utilized for quality assessment and is provided online in Appendix 2 (Slim et al., 2003). Any disagreements were resolved by consensus or consultation of a third reviewer (JWC).

IPD were extracted from the included studies. If data were unavailable, authors were contacted and raw data were requested. In additional data provided by authors, angular deformities were measured on original radiographs. These additional measurements were added to the data sheet. Intra-class correlation range was determined. Van Geenen et al. anonymously supplied radiographs of 19 eligible participants, in which we measured the angular deformities with an intra-class correlation range of 0.91–0.99 (van Geenen and Besselaar, 2007). Walenkamp et al. also provided raw data, supplied online in Appendix 3 (Walenkamp et al., 2015). Within the included studies, participants’ raw data were screened and only participants meeting the inclusion criteria were included in our meta-analysis. Reasons for exclusion involved other indications for corrective osteotomy than deficit in ROM; an age at trauma over 16 years of age and/or an age at osteotomy over 18 years of age. Data extraction was verified by the second reviewer. The available IPD were assembled and analysed as if they were results from one study.

We performed one-way analysis of variance (ANOVA) with clinically relevant subgroups for each factor we investigated. Subgroups were created for: (1) age at trauma (younger than 10 years versus 10 years and older); (2) age at corrective osteotomy (younger than 13 years versus 13 years and older); (3) time from trauma until corrective osteotomy (within 1  year after trauma versus 1 year after trauma or more); (4) level of malunion (in the proximal, middle or distal third); (5) severity of angular deformity (under 20° versus 20° or more); (6) type of corrective osteotomy (three-dimensional computer-assisted corrective osteotomy versus conventional corrective osteotomy using two-dimensional radiographic planning); and (7) pre-operative complaint (predominant deficit in pronation versus predominant deficit in supination). Performing a corrective osteotomy within 1 year after trauma was defined as early management, whereas more than 1 year was defined as late management (Trousdale and Linscheid, 1995). Subgroups dividing age at trauma were set at below or above 10 years in accordance with an earlier study (van Geenen and Besselaar, 2007). We set the cut-off for age at osteotomy at below or above 13 years of age, due to a mean time from trauma until osteotomy of 3 years in a previous study (van Geenen and Besselaar, 2007). Severity of angulation was subdivided at below or above 20°, because in a cadaveric study, there was a statistically significant and functionally important loss of forearm rotation if angulation exceeded 20° (Matthews et al., 1982).

Next, multivariate regression analysis was performed to study the effect of the various factors on the gain in ROM after corrective osteotomy, using a stepwise backward procedure. We reported medians and interquartile range (IQR) for non-parametric variables, and means and standard deviations (SD) for normally distributed variables. The 95% confidence intervals (CI) were calculated using the formula: χ SD 1.96 (σ/√n), with χ = mean; a confidence coefficient of 1.96 for a confidence level of 95%; σ = standard deviation of sample; (square root of) *n* = sample size. *P*-values < 0.05 were considered statistically significant.

## Results

Our search resulted in 1423 potentially eligible studies, of which 22 full-text articles were analysed for eligibility. A total of 12 studies met the inclusion criteria (Boeckers et al., 2014; Chia et al., 2011; Kataoka et al., 2013; Meier et al., 2003, 2004; Miyake et al., 2012; Murase et al., 2008; Nagy et al., 2008; Price and Knapp, 2006; Trousdale and Linscheid, 1995; van Geenen and Besselaar, 2007; Walenkamp et al., 2015). Two studies by Meier et al. contained duplicate participants (Meier et al., 2003, 2004). Therefore, 11 studies with IPD were included in the IPD meta-analysis, shown in the flow diagram in Figure S1 (online supplement). Assessment of methodological quality in the included studies is provided in Table 1. The included studies contained 158 participants who were treated for a symptomatic radius or both-bone forearm malunion by corrective osteotomy, of which 71 participants met the inclusion criteria. The participants fulfilling the eligibility criteria are reported in Table 2 with notes on the reasons for exclusion. The most common reasons for exclusion were failure to match the inclusion criteria for age, or due to alternative indications for corrective osteotomy, such as a painful distal radio-ulnar joint, cosmetic appearance or a congenital deformity. Details on degree of radiographic angular deformity were provided in 49 out of 71 participants. Corrective osteotomies using three-dimensional computer-assisted techniques were performed in four out of 11 studies. A summary of characteristics and outcomes of the individual studies is presented in Table 3, with medians for age at trauma, time until osteotomy and duration of follow-up and mean functional and radiographic measurements. A full overview of extracted IPD is supplemented online in Appendix 3.

**Table 1.** MINORS methodological quality.

Study	Clear aim	Inclusion patients	Collection data	Appropriate end points	Assessment end points	Follow-up period	Loss to follow-up	Calculation study size	Total
Trousdale	1	2	0	2	0	2	1	0	8
Meier	2	2	0	2	0	2	2	0	10
Price	2	2	0	2	0	2	2	0	10
Van Geenen	1	2	0	2	0	2	2	0	10
Murase	2	2	2	2	1	2	2	0	13
Nagy	2	0	0	2	1	2	2	0	9
Chia	2	2	0	2	0	2	2	0	10
Miyake	2	2	0	2	1	2	1	0	10
Kataoka	2	2	0	2	1	2	2	0	11
Boeckers	1	2	0	2	0	1	2	0	8
Walenkamp	2	2	0	2	1	1	0	0	8

The items are scored 0 (not reported), 1 (reported but inadequate) or 2 (reported and adequate).

**Table 2.** Extraction of IPD.

Year	Study	Eligible participants	Total participants	Design	Excluded (participant number)	Reasons for exclusion^a^
1995	Trousdale	14	27	Retrospective	3,6,10,14,15,19,21–27	Age, other
2003	Meier (GER)	6	14	Retrospective	All but 4,8–11,14	Other, age, TUO,
2006	Price	9	9	Retrospective	None	–
2007	van Geenen	17	21	Retrospective	6,12,20,21	TUO, FU, age
2008	Murase^b^	4	22	Prospective	All but 5,8,9,14	Age
2008	Nagy	7	17	Retrospective	2,6,7, 11–17	Age, other
2011	Chia	1	6	Retrospective	All but 4	Age
2012	Miyake^b^	9	20	Retrospective	1,4–7,13,15–18,20	Age
2013	Kataoka^b^	1	9	Retrospective	All but 5	Age at trauma
2014	Boeckers (GER)	1	5	Retrospective	All but 4	FU, TUO
2015	Walenkamp^b^	2	8	Retrospective	All but 4,8	Age, other
2016	Current study	71	158	Meta-analysis	—	—

a Age: age at trauma above 16 and/or osteotomy above 18 years; TUO: time until osteotomy <6 weeks; FU: follow-up <6 m.

b Three-dimensional computer-assisted corrective osteotomy; GER: German.

**Table 3.** Study characteristics.

Year	Study	Age at trauma	Years until osteotomy	Months follow-up	Angulation	Pre-op ROM	ROM at FU	Gain in ROM	Complications
1995	Trousdale	11	3	61	NR	78°	132°	53°	5
2003	Meier	11	1	13	NR	76°	159°	83°	1
2006	Price	7	1	22	31°	63°	165°	102°	2
2007	van Geenen	9	2	26	30°	34°	120°	86°	1
2008	Murase^a^	11	4	22	18°	51°	144°	93°	1
2008	Nagy	12	4	41	18°	86°	137°	51°	0
2011	Chia	14	1	42	20°	130°	175°	45°	0
2012	Miyake^a^	11	4	30	22°	57°	146°	90°	0
2013	Kataoka^a^	4	7	22	35°	70°	130°	60°	0
2014	Boeckers	13	0,1	7	NR	90°	180°	90°	0
2015	Walenkamp^a^	1	4	18	14°	103°	158°	55°	0
2016	Current study	11	1,0	29	25°	63°	140°	77°	10

a Three-dimensional computer-assisted corrective osteotomy.

ROM: range of motion; FU: follow-up; NR: not reported.

### Characteristics of IPD

The majority of participants were male (61%). Fractures of both forearm bones were seen in 45 out of 71 participants (63%). The malunions were located in the proximal third in 15 participants (21%), the middle third in 44 (62%) and the distal third in 12 (17%). Included participants had a median age at trauma of 11 years (IQR 8 to 13). Median age at corrective osteotomy was 13 years (IQR 11 to 16). Median time from trauma until osteotomy was 12 months (IQR 6 to 48). Functional outcome at final follow-up was measured at a median time of 29 months (IQR 16 to 37) after corrective osteotomy. As pre-operative complaint, 20 predominately had a deficit in pronation, 34 predominately had a deficit in supination and 17 had a similar deficit in both pro- and supination. Corrective osteotomies using three-dimensional computer-assisted techniques were performed in 16 participants, whereas 55 participants underwent conventional corrective osteotomy using two-dimensional pre-operative planning with standard radiographs. There was a complication rate of 14%, which primarily consisted of superficial infection or transient dysesthesia of the radial sensory nerve. There were no major complications.

### Results of syntheses

Overall, there was a mean pre-operative forearm rotation of 63° (95% CI: 55° to 70°). At final follow-up, there was a mean forearm rotation of 140° (132° to 148°) indicating that corrective osteotomy provided a mean gain in forearm rotation of 77° (68° to 86°). Results of one-way ANOVA are presented in Table 4 showing comparisons of outcomes of clinically relevant subgroups with regards to our primary outcome, the gain in forearm rotation.

**Table 4.** ANOVA: effect of factors on gain in pro-supination.

Factor		*N*	Pre-op ROM (95% CI)	*P* =	ROM at FU (95% CI)	*P* =	Gain in ROM (95% CI)	*P* =
Age at trauma	<10 years	28	57° (46° to 69°)	0.23	132° (118° to 145°)	0.11	74° (58° to 90°)	0.64
	⩾10 years	43	66° (57° to 77°)		145° (135° to 156°)		79° (67° to 90°)	
Age at osteotomy	< 13 years	33	53° (42° to 65°)	0.013	141° (128° to 154°)	0.87	87° (74° to 101°)	0.031
	⩾13 years	38	71° (62° to 81°)		139° (128° to 150°)		68° (56° to 80°)	
Time until osteotomy	< 1 year	36	61° (50° to 73°)	0.69	154° (144° to 164°)	<0.001	93° (80° to 106°)	<0.001
	⩾ 1 year	35	64° (55° to 74°)		125° (114° to 137°)		61° (50° to 72°)	
Location of malunion	Proximal	15	50° (32° to 68°)	0.08	113° (96° to 130°)	0.003	63° (43° to 84°)	0.16
	Middle	44	63° (54° to 73°)		147° (137° to 157°)		84° (73° to 95°)	
	Distal	12	63° (55° to 70°)		146° (126° to 166°)		69° (43° to 95°)	
Boned malunited	Single	26	67° (55° to 80°)	0.40	142° (129° to 155°)	0.66	75° (60° to 90°)	0.77
	Both-bone	45	60° (51° to 70°)		138° (128° to 149°)		78° (66° to 90°)	
Angulation	<20°	18	70° (54° to 86°)	0.030	129° (109° to 149°)	0.08	59° (45° to 74°)	<0.001
	⩾20°	31	50° (38° to 61°)		146° (136° to 156°)		97° (85° to 108°)	
Technique	Conventional	55	63° (54° to 72°)	0.88	138° (128° to 148°)	0.43	75° (64° to 85°)	0.41
	3-D assisted	16	62° (48° to 76°)		146° (129° to 162°)		84° (64° to 104°)	
Complaint	Pro-deficit	34	65° (54° to 76°)	0.18	136° (123° to 149°)	0.74	71° (58° to 83°)	0.42
	Sup- deficit	20	77° (64° to 90°)		139° (124° to 154°)		63° (45° to 80°)	
Total		71	63° (55° to 70°)		140° (132° to 148°)		77° (68° to 86°)	

ROM: range of motion; CI: confidence intervals; FU: follow-up; 3-D: three-dimensional.

We found the following statistically significant differences during ANOVA: children who underwent corrective osteotomy at an age younger than 13 years had a mean gain of 87° (74° to 101°) in forearm rotation, versus a mean gain of 68° (56° to 80°) in children aged 13 years and older (*p* = 0.031). Participants who underwent corrective osteotomy within 1 year after trauma gained 93° (80° to 106°) versus 61° (50° to 72°) in those who underwent osteotomy more than 1 year after trauma (*p* < 0.001). Participants who had an angular deformity of less than 20° had a mean gain in forearm rotation after corrective osteotomy of 59° (45° to 74°) versus a mean gain of 97° (85° to 108°) in those with 20° of angulation or more (*p* < 0.001).

ANOVA revealed that level of malunion was not statistically significantly associated with a higher gain in pronosupination. An additional independent sample’s *T*-test was performed comparing malunions located in the middle third versus malunions located in the proximal and distal third, revealed a gain of, respectively, 84° (72° to 95°)versus 66° (51° to 81°) in pronosupination (*p* = 0.057).

Multi-variate regression analysis revealed that a shorter time until osteotomy, a greater angular deformity and the use of three-dimensional computer-assisted techniques were factors associated with a greater gain in forearm rotation (*p*-values are, respectively, 0.002, 0.044 and 0.042). The results of multiple regression analysis, including Beta values and standard errors, are presented in Table 5. There was an R square of 0.35.

**Table 5.** Multiple regression analysis.

Model	Unstandardized coefficients		
	B	Std error	Significance
(Constant)	62.1	15.1	0.000
Months until osteotomy	−0.45	0.14	0.002
Angulation	0.95	0.46	0.044
Three-dimensional techniques	24.3	11.6	0.042

R square: 0.345, adjusted R square 0.302.

## Discussion

In the literature, recommendations on indications for corrective osteotomy have been based on age and location of the malunion, severity of functional impairment and/or severity of angular deformity. Prommersberger et al. stated that in the case of functional disability, there is an indication for corrective osteotomy over the age over 12 in malunion of a fracture located in the distal third, and over the age of 5 in gross deformity of fractures to the midshaft of the forearm (Prommersberger and Lanz, 2000). Others stated that an early corrective osteotomy is justified in patients with an established malunion with considerable functional impairment (pronosupination of less than 50%–60% of normal) (van Geenen and Besselaar, 2007). Price et al. recommended to perform corrective osteotomy in forearm shaft malunions with angulations of greater than 30° as soon as possible; and to wait at least 6 months in malunions with angulations ranging from 20°–30°, because the greatest amount of remodelling occurs in the first 6 months (Price and Knapp, 2006).

Previous studies have generally suggested that children gain more in ROM if corrective osteotomy is performed at a younger age. It is suggested that this is due to the potential for residual bone deformities to improve with additional skeletal growth (Nagy et al., 2008; van Geenen and Besselaar, 2007). In our IPD meta-analysis, ANOVA revealed that both a younger age at osteotomy and a shorter time until osteotomy were associated with a better functional outcome. Logically, there was an overlap between these two groups, because participants with a shorter time until osteotomy often had a younger age at osteotomy than participants with a longer time until osteotomy. However, multiple regression analysis, which simultaneously studies the relationship between multiple factors, revealed that a shorter time until osteotomy is associated with a higher functional outcome, and this achieved statistical significance. This was not the case with a younger age at osteotomy.

Previous studies also found that a longer time from trauma until osteotomy compromised functional gain, which was thought to be the result of secondary joint changes and soft-tissue contractures (Trousdale and Linscheid, 1995; van Geenen and Besselaar, 2007). However, the presence of these soft-tissue contractures is yet to be proven. In a previous study, children who had a persisting deficit in pronosupination exceeding 40° at a follow-up beyond 6 months after fracture of both forearm bones underwent magnetic resonance imaging (MRI) analysis, which did not reveal contractures of the interosseous membrane (Colaris et al., 2014b). The question remains whether the contractures did not exist, or whether they were not detectable on MRI. In our IPD meta-analysis, a shorter time until osteotomy was the most decisive factor in predicting a superior functional outcome, which does suggest a role of secondary joint changes and soft-tissue contractures.

One previous study analysed the effect of location of the malunion and the outcome after corrective osteotomy and found no statistically significant effect (van Geenen and Besselaar, 2007). In our IPD meta-analysis, we saw a moderate trend for the most favourable results after corrective osteotomies for malunions located in the middle third and the poorest results in proximal malunions; this did not achieve statistical significance (*p* = 0.057). Although a recent cadaveric study showed that dorsal tilt up to 30° did not lead to any significant restriction in forearm pronosupination (Bronstein et al., 2014), most studies have shown that angular deformity plays an important role in the limitation of forearm rotation (Colaris et al., 2014a; Dumont et al., 2002; Matthews et al., 1982; Sarmiento et al., 1992; Tarr et al., 1984). In our IPD meta-analysis, greater pre-operative angulation was associated with superior functional outcomes after corrective osteotomy. Moreover, a previous study advocated that improvement in ROM was greater in those who predominately had a supination deficit as pre-operative complaint (Nagy et al., 2008). This was not supported by our IPD meta-analysis.

In a previous study, computer-assisted three-dimensional planning was found to improve functional results in patients with symptomatic radius malunions (Vroemen et al., 2013; Walenkamp et al., 2015). In our meta-analysis, the use of three-dimensional computer-assisted techniques also had a statistically significant effect on functional outcome.

The main strength of this study is the access to IPD, which provided the opportunity to analyse a higher number of patients, resulting in several recommendations. A weakness of this meta-analysis is that the majority of the included studies were of retrospective nature. Furthermore, patient-reported outcome measures were not reported in the majority of included studies. Also, there were no control groups, so there is no possibility to compare functional outcomes with those who did not undergo a corrective osteotomy for their post-traumatic forearm malunion. Lastly, we included isolated radius fractures as well as fractures of both forearm bones in our IPD meta-analysis. However, we found no statistically significant difference in the gain of function after corrective osteotomy when comparing isolated radius and both-bone forearm fractures.

This meta-analysis of IPD provides recommendations that can facilitate decision making when considering corrective osteotomy for malunited paediatric fractures of the radius or both forearm bones. Based on this meta-analysis, predictors of a superior functional outcome are: an interval between trauma and corrective osteotomy of less than 1 year; an angular deformity greater than 20°; and the use of three-dimensional computer-assisted techniques.

## Supplementary Material

Supplementary material

Supplementary material
