# Supplementary material for: Factors determining outcome of corrective osteotomy for malunited paediatric forearm fractures: a systematic review and meta-analysis
Source: J Hand Surg Eur Vol. 2017 Jun 15;42(8):810–6. doi: 10.1177/1753193417711684 (PMC5598749; doi:10.1177/1753193417711684)
Supplement: Supplementary material [file Supplementary_Appendix.docx]

**Appendix 1. Database search strategy**

| **Database search strategy Medline and Embase:**  ('forearm fracture'/de OR 'radius fracture'/de OR 'ulna fracture'/de OR ((forearm* OR antebrachi* OR radius OR radial OR 'lower arm' OR ulna*) NEAR/11 fractur*):ab,ti) AND (osteotomy/de OR (osteotom* OR correcti*):ab,ti) AND (child/exp OR newborn/exp OR pediatrics/exp OR childhood/exp OR 'child growth'/de OR 'child health'/de OR 'child health care'/exp OR 'child care'/exp OR 'pediatric ward'/de OR 'pediatric hospital'/de OR adolescent/exp OR adolescence/exp OR (infan* OR newborn* OR (new NEXT/1 born*) OR baby OR babies OR neonat* OR child* OR kid OR kids OR toddler* OR teen* OR boy OR boys OR girl OR girls OR minors OR underag* OR juvenil* OR youth* OR kindergar* OR puber* OR pubescen* OR prepubescen* OR prepubert* OR pediatric* OR paediatric* OR school* OR preschool* OR highschool* OR adolescen*):ab,ti)  **Database search strategy Cochrane, Web-of-Science, Scopus, Cinahl, Pubmed Publisher and Google Scholar:**  (((forearm* OR antebrachi* OR radius OR radial OR 'lower arm' OR ulna*) NEAR/11 fractur*):ab,ti) AND (osteotom* OR correcti*):ab,ti AND ((infan* OR newborn* OR (new NEXT/1 born*) OR baby OR babies OR neonat* OR child* OR kid OR kids OR toddler* OR teen* OR boy OR boys OR girl OR girls OR minors OR underag* OR juvenil* OR youth* OR kindergar* OR puber* OR pubescen* OR prepubescen* OR prepubert* OR pediatric* OR paediatric* OR school* OR preschool* OR highschool* OR adolescen*):ab,ti) |
| --- |

**Appendix 2. MINORS methodological quality assessment instrument**

| **Methodological index for non-randomized studies** | **Score** |
| --- | --- |
| 1. A clearly stated aim: the question addressed should be precise and relevant in the light of available literature |  |
| 1. Inclusion of consecutive patients: all patients potentially fit for inclusion (satisfying the criteria for inclusion) have been included in the study during the study period (no exclusion or details about the reasons for exclusion) |  |
| 1. Prospective collection of data: data were collected according to a protocol established before the beginning of the study |  |
| 1. Endpoints appropriate to the aim of the study: unambiguous explanation of the criteria used to evaluate the main outcome which should be in accordance with the question addressed by the study. Also, the endpoints should be assessed on an intention-to-treat basis. |  |
| 1. Unbiased assessment of the study endpoint: blind evaluation of objective endpoints and double-blind evaluation of subjective endpoints. Otherwise the reasons for not blinding should be stated |  |
| 1. Follow-up period appropriate to the aim of the study: the follow-up should be sufficiently long to allow the assessment of the main endpoint and possible adverse events |  |
| 1. Loss to follow up less than 5%: all patients should be included in the follow up. Otherwise, the proportion lost to follow up should not exceed the proportion experiencing the major endpoint |  |
| 1. Prospective calculation of the study size: information of the size of detectable difference of interest with a calculation of 95% confidence interval, according to the expected incidence of the outcome event, and information about the level for statistical significance and estimates of power when comparing the outcomes |  |
| 1. Additional criteria in the case of comparative study |  |
| 1. An adequate control group: having a gold standard diagnostic test or therapeutic intervention recognized as the optimal intervention according to the available published data |  |
| 1. Contemporary groups: control and studied group should be managed during the same time period (no historical comparison) |  |
| 1. Baseline equivalence of groups: the groups should be similar regarding the criteria other than the studied endpoints. Absence of confounding factors that could bias the interpretation of the results |  |
| 1. Adequate statistical analyses: whether the statistics were in accordance with the type of study with calculation of confidence intervals or relative risk |  |
| † The items are scored 0 (not reported), 1 (reported but inadequate) or 2 (reported and adequate).  The global ideal score being 16 for non-comparative studies and 24 for comparative studies. | |

**Appendix 3. Overview of extracted individual participant data**

| Year | Study | N | Sex | Fracture location | Both-bone fracture | Angulation | Age trauma | Age osteotomy | Years until osteotomy | Follow-up (months) | Pre-op pronation | Pre-op supination | Pre-operative ROM | Pronation at FU | Supination at FU | Pro-sup at FU | Gain in ROM | Use of 3-D technique | Complication | Supplied on request |
| --- | --- | --- | --- | --- | --- | --- | --- | --- | --- | --- | --- | --- | --- | --- | --- | --- | --- | --- | --- | --- |
| 1995 | Trousdale | 1 | F | Distal | Y | NR | 13 | 13 | 0.7 | 35 | 72 | 30 | 102 | 85 | 85 | 170 | 68 | N | N | N |
| 1995 | Trousdale | 2 | M | Distal | Y | NR | 16 | 17 | 1 | 180 | 20 | 75 | 95 | 60 | 70 | 130 | 35 | N | Y | N |
| 1995 | Trousdale | 4 | F | Proximal | N | NR | 12 | 13 | 0.4 | 19 | 20 | 90 | 110 | 45 | 85 | 130 | 20 | N | N | N |
| 1995 | Trousdale | 5 | M | Middle | Y | NR | 9 | 10 | 0.4 | 84 | 10 | 10 | 20 | 90 | 90 | 180 | 160 | N | Y | N |
| 1995 | Trousdale | 7 | F | Proximal | Y | NR | 16 | 17 | 1 | 34 | 65 | 15 | 80 | 85 | 85 | 170 | 90 | N | N | N |
| 1995 | Trousdale | 8 | M | Distal | N | NR | 13 | 13 | 0.2 | 31 | 15 | 30 | 45 | 85 | 70 | 155 | 110 | N | N | N |
| 1995 | Trousdale | 9 | F | Middle | N | NR | 16 | 17 | 0.7 | 49 | 75 | 40 | 115 | 75 | 75 | 150 | 35 | N | Y | N |
| 1995 | Trousdale | 11 | M | Distal | Y | NR | 8 | 10 | 2 | 15 | 90 | 0 | 90 | 85 | 40 | 125 | 35 | N | N | N |
| 1995 | Trousdale | 12 | M | Distal | Y | NR | 7 | 18 | 11 | 65 | 80 | 5 | 85 | 70 | 5 | 75 | -10 | N | Y | N |
| 1995 | Trousdale | 13 | M | Middle | Y | NR | 11 | 13 | 2 | 14 | 0 | 85 | 85 | 90 | 90 | 180 | 95 | N | N | N |
| 1995 | Trousdale | 16 | M | Proximal | N | NR | 11 | 17 | 6 | 120 | 20 | 50 | 70 | 50 | -5 | 45 | -25 | N | Y | N |
| 1995 | Trousdale | 17 | M | Proximal | Y | NR | 6 | 12 | 6 | 80 | 45 | 0 | 45 | 60 | 35 | 95 | 50 | N | N | N |
| 1995 | Trousdale | 18 | M | Proximal | N | NR | 4 | 9 | 6 | 90 | 85 | 15 | 100 | 75 | 45 | 125 | 25 | N | N | N |
| 1995 | Trousdale | 20 | F | Proximal | Y | NR | 12 | 16 | 4 | 36 | 10 | 45 | 55 | 85 | 30 | 115 | 60 | N | N | N |
| 2003 | Meier | 4 | F | Distal | N | NR | 14 | 14 | 0.4 | 19 | 70 | 30 | 100 | 100 | 90 | 190 | 90 | N | N | N |
| 2003 | Meier | 8 | M | Middle | NR | NR | 5 | 6 | 0.8 | 12 | 50 | 30 | 80 | 45 | 70 | 115 | 35 | N | N | N |
| 2003 | Meier | 9 | F | Middle | NR | NR | 11 | 14 | 3 | 8 | 40 | 30 | 70 | 80 | 80 | 160 | 90 | N | Y | N |
| 2003 | Meier | 10 | F | Middle | NR | NR | 10 | 10 | 0.2 | 17 | 20 | 20 | 40 | 70 | 90 | 160 | 120 | N | N | N |
| 2003 | Meier | 11 | F | Middle | NR | NR | 10 | 12 | 2 | 6 | 0 | 90 | 90 | 90 | 70 | 160 | 70 | N | N | N |
| 2003 | Meier | 14 | M | Middle | NR | NR | 15 | 16 | 0.3 | 16 | 45 | 30 | 75 | 90 | 80 | 170 | 95 | N | N | N |
| 2006 | Price | 1 | M | Middle | Y | 32 | 5 | 6 | 0.8 | 7 | 20 | 0 | 20 | 90 | 90 | 180 | 160 | N | N | N |
| 2006 | Price | 2 | M | Middle | Y | 45 | 6 | 7 | 0.6 | 16 | 45 | 25 | 70 | 50 | 90 | 140 | 70 | N | N | N |
| 2006 | Price | 3 | M | Middle | Y | 30 | 5 | 5 | 0.3 | 6 | 30 | 30 | 60 | 80 | 90 | 170 | 110 | N | N | N |
| 2006 | Price | 4 | F | Middle | Y | 35 | 10 | 10 | 0.6 | 9 | 20 | 20 | 40 | 70 | 90 | 160 | 120 | N | N | N |
| 2006 | Price | 5 | M | Middle | N | 34 | 5 | 6 | 0.8 | 20 | 20 | 20 | 40 | 90 | 90 | 180 | 140 | N | N | N |
| 2006 | Price | 6 | F | Middle | N | 30 | 5 | 5 | 0.3 | 60 | 45 | 45 | 90 | 70 | 90 | 160 | 70 | N | N | N |
| 2006 | Price | 7 | F | Middle | Y | 15 | 11 | 12 | 1 | 58 | 30 | -10 | 20 | 45 | 90 | 135 | 115 | N | N | N |
| 2006 | Price | 8 | M | Middle | N | 25 | 8 | 9 | 0.3 | 12 | 45 | 90 | 135 | 90 | 90 | 180 | 45 | N | Y | N |
| 2006 | Price | 9 | M | Middle | Y | 30 | 5 | 5 | 0.3 | 13 | 0 | 90 | 90 | 90 | 90 | 180 | 90 | N | Y | N |
| 2007 | van Geenen | 1 | F | Middle | Y | NR | 9 | 11 | 2 | 59 | 20 | 10 | 30 | 45 | 45 | 90 | 60 | N | N | Y |
| 2007 | van Geenen | 2 | M | Distal | N | 40 | 6 | 7 | 0.3 | 9 | 40 | 0 | 40 | 70 | 80 | 150 | 110 | N | N | Y |
| 2007 | van Geenen | 3 | F | Middle | Y | 40 | 2 | 7 | 5 | 12 | 90 | -60 | 30 | 45 | 80 | 125 | 95 | N | N | Y |
| 2007 | van Geenen | 4 | F | Distal | N | 23 | 4 | 4 | 0.2 | 12 | 0 | 35 | 35 | 70 | 80 | 150 | 115 | N | N | Y |
| 2007 | van Geenen | 5 | M | Proximal | N | 45 | 10 | 13 | 3 | 51 | 10 | 10 | 20 | 45 | 60 | 105 | 85 | N | N | Y |
| 2007 | van Geenen | 7 | F | Proximal | N | 13 | 5 | 12 | 7 | 33 | 60 | -30 | 30 | 70 | 30 | 100 | 70 | N | N | Y |
| 2007 | van Geenen | 8 | M | Middle | Y | 10 | 13 | 16 | 3 | 51 | 20 | 70 | 90 | 60 | 90 | 150 | 60 | N | N | Y |
| 2007 | van Geenen | 9 | M | Distal | N | 35 | 15 | 15 | 0.2 | 17 | 20 | 40 | 60 | 70 | 80 | 150 | 90 | N | N | Y |
| 2007 | van Geenen | 10 | F | Proximal | Y | 30 | 10 | 14 | 5 | 21 | 10 | 0 | 10 | 50 | 30 | 80 | 70 | N | Y | Y |
| 2007 | van Geenen | 11 | M | Middle | Y | 33 | 11 | 11 | 0.2 | 23 | 5 | 5 | 10 | 70 | 80 | 150 | 140 | N | N | Y |
| 2007 | van Geenen | 13 | M | Proximal | N | 20 | 9 | 11 | 2 | 22 | 5 | 20 | 25 | 70 | 20 | 90 | 65 | N | N | Y |
| 2007 | van Geenen | 14 | F | Proximal | Y | 45 | 8 | 8 | 0.3 | 26 | 20 | 20 | 40 | 60 | 80 | 140 | 100 | N | N | Y |
| 2007 | van Geenen | 15 | F | Proximal | Y | 23 | 8 | 8 | 0.7 | 22 | 20 | 10 | 30 | 60 | 80 | 140 | 110 | N | N | Y |
| 2007 | van Geenen | 16 | M | Middle | Y | 15 | 13 | 14 | 0.5 | 24 | 10 | -10 | 0 | 10 | 10 | 20 | 20 | N | N | Y |
| 2007 | van Geenen | 17 | M | Proximal | N | 20 | 12 | 13 | 2 | 21 | 0 | 45 | 45 | 60 | 80 | 140 | 95 | N | N | Y |
| 2007 | van Geenen | 18 | M | Distal | Y | 20 | 14 | 15 | 2 | 19 | 45 | 40 | 85 | 85 | 80 | 165 | 80 | N | N | Y |
| 2007 | van Geenen | 19 | F | Proximal | Y | 27 | 11 | 12 | 1 | 15 | 45 | -45 | 0 | 80 | 20 | 100 | 100 | N | N | Y |
| 2008 | Nagy | 1 | F | Middle | Y | 15 | 12 | 18 | 7 | 119 | 15 | 75 | 90 | 70 | 80 | 150 | 60 | N | N | N |
| 2008 | Nagy | 3 | M | Middle | Y | 20 | 14 | 16 | 2 | 12 | 10 | 75 | 85 | 60 | 80 | 140 | 55 | N | N | N |
| 2008 | Nagy | 4 | F | Middle | Y | 16 | 13 | 14 | 0.9 | 6 | 5 | 90 | 95 | 50 | 90 | 140 | 45 | N | N | N |
| 2008 | Nagy | 5 | F | Distal | Y | 16 | 15 | 16 | 2 | 37 | 40 | 60 | 100 | 65 | 50 | 115 | 15 | N | N | N |
| 2008 | Nagy | 8 | M | Middle | Y | 16 | 14 | 18 | 4 | 46 | 70 | 0 | 70 | 60 | 90 | 150 | 80 | N | N | N |
| 2008 | Nagy | 9 | M | Middle | Y | 30 | 11 | 13 | 2 | 18 | 90 | 10 | 100 | 80 | 90 | 170 | 70 | N | N | N |
| 2008 | Nagy | 10 | M | Middle | Y | 10 | 7 | 18 | 11 | 46 | 60 | 0 | 60 | 25 | 70 | 95 | 35 | N | N | N |
| 2008 | Murase | 5 | F | Middle | Y | 12 | 8 | 16 | 8 | 24 | 80 | -30 | 50 | 80 | 10 | 90 | 40 | Y | Y | N |
| 2008 | Murase | 8 | M | Middle | Y | 33 | 12 | 12 | 0.5 | 22 | 10 | 15 | 25 | 95 | 80 | 175 | 150 | Y | N | N |
| 2008 | Murase | 9 | M | Middle | N | 22 | 13 | 14 | 0.8 | 23 | 60 | -20 | 40 | 70 | 70 | 140 | 100 | Y | N | N |
| 2008 | Murase | 14 | M | Middle | Y | 6 | 11 | 18 | 7 | 14 | 80 | 10 | 90 | 90 | 80 | 170 | 80 | Y | N | N |
| 2011 | Chia | 4 | M | Proximal | N | 20 | 14 | 16 | 1.3 | 42 | 65 | 65 | 130 | 90 | 85 | 175 | 45 | N | N | N |
| 2012 | Miyake | 2 | M | Middle | N | 22 | 12 | 13 | 0.8 | 24 | 60 | -20 | 40 | 70 | 70 | 140 | 100 | Y | N | N |
| 2012 | Miyake | 3 | F | Middle | Y | 12 | 7 | 15 | 8 | 24 | 80 | -30 | 50 | 80 | 0 | 80 | 30 | Y | N | N |
| 2012 | Miyake | 8 | M | Middle | Y | 33 | 11 | 12 | 0.4 | 24 | 10 | 15 | 25 | 80 | 95 | 175 | 150 | Y | N | N |
| 2012 | Miyake | 9 | M | Middle | Y | 16 | 13 | 18 | 6 | 33 | 70 | 20 | 90 | 70 | 90 | 160 | 70 | Y | N | N |
| 2012 | Miyake | 10 | M | Middle | N | 14 | 16 | 16 | 0.7 | 48 | 60 | 10 | 70 | 70 | 80 | 150 | 80 | Y | N | N |
| 2012 | Miyake | 11 | F | Middle | Y | 13 | 11 | 16 | 5 | 37 | 60 | 0 | 60 | 90 | 90 | 180 | 120 | Y | N | N |
| 2012 | Miyake | 12 | M | Middle | Y | 23 | 13 | 17 | 4 | 24 | 0 | 45 | 45 | 70 | 90 | 160 | 115 | Y | N | N |
| 2012 | Miyake | 14 | M | Middle | Y | 27 | 10 | 11 | 0.8 | 32 | 60 | 0 | 60 | 80 | 80 | 160 | 100 | Y | N | N |
| 2012 | Miyake | 19 | F | Middle | Y | 35 | 3 | 11 | 8 | 28 | 90 | -20 | 70 | 90 | 20 | 110 | 40 | Y | N | N |
| 2013 | Kataoka | 5 | F | Middle | Y | 35 | 4 | 11 | 7 | 22 | 90 | -20 | 70 | 90 | 40 | 130 | 60 | Y | N | N |
| 2014 | Boeckers | 4 | M | Distal | N | NR | 13 | 13 | 0.1 | 7 | 90 | 0 | 90 | 90 | 90 | 180 | 90 | N | N | N |
| 2015 | Walenkamp | 4 | M | Middle | Y | 12 | 14 | 18 | 4 | 13 | 40 | 40 | 80 | 60 | 75 | 135 | 55 | Y | N | Y |
| 2015 | Walenkamp | 8 | M | Middle | Y | 16 | 12 | 13 | 0.8 | 23 | 90 | 35 | 125 | 90 | 90 | 180 | 55 | Y | N | Y |
| NR = Not reported, M = Male, F = Female, Y = Yes, N = No. | | | | | | | | | | | | | | | | | | | | |
