# Supplementary material for: Factors determining outcome of corrective osteotomy for malunited paediatric forearm fractures: a systematic review and meta-analysis
Source: J Hand Surg Eur Vol. 2017 Jun 15;42(8):810–6. doi: 10.1177/1753193417711684 (PMC5598749; doi:10.1177/1753193417711684)
Supplement: Supplementary material [file Supplementary_Figure_S1.docx]

**Figure 1. PRISMA flow diagram**

12 Studies for which individual participant data provided

158 participants for whom data were provided

0 studies for which aggregate data were available

0 Studies for which IPD were not provided

**Aggregate data:**

0 studies included in analysis

**Individual participant data:**
12 studies included in analysis:

71 participants included

(87 participants excluded)

0 Eligible studies for which IPD were not sought

Analyzed data

## ed

Available data

## ed

Obtaining data

## ed

Screening

Eligibility

Identification

1 Additional record identified through cross-reference checking
(Walenkamp et al.)

**1422** Records identified through database searching

650 Records after duplicates removed

650 Records screened

628 Records excluded based on title and abstract

10 Full-text articles excluded

12 Studies for which individual participant data were sought

22 Full-text articles assessed for eligibility
